# Coronavirus Disease 2019 Outcomes in French Nursing Homes That Implemented Staff Confinement With Residents

**DOI:** 10.1001/jamanetworkopen.2020.17533

**Published:** 2020-08-13

**Authors:** Joël Belmin, Nathavy Um-Din, Cristiano Donadio, Maurizio Magri, Quoc Duy Nghiem, Bruno Oquendo, Sylvie Pariel, Carmelo Lafuente-Lafuente

**Affiliations:** 1 Service Universitaire de Gériatrie, Hôpital Charles Foix, Groupe Hospitalier APHP Sorbonne Université, Ivry-sur-Seine, France; 2 Faculté de Médecine Sorbonne Médecine, Paris, France

## Abstract

**Importance:**

Coronavirus disease 2019 (COVID-19) is a major threat to nursing homes. During the COVID-19 pandemic wave that hit France in March and April 2020, staff members of some French nursing homes decided to confine themselves with their residents on a voluntary basis to reduce the risk of entry of the severe acute respiratory syndrome coronavirus 2 into the facility.

**Objective:**

To investigate COVID-19–related outcomes in French nursing homes that implemented voluntary staff confinement with residents.

**Design, Setting, and Participants:**

This retrospective cohort study was conducted in French nursing homes from March 1 to May 11, 2020. Participants included residents and staff members of the nursing homes where staff participated in voluntary self-confinement as well as those of the facilities for elderly people where staff did not practice self-confinement. Rates of COVID-19 cases and mortality in the cohort of nursing homes with self confinement were compared with those derived from a population-based survey of nursing homes conducted by French health authorities.

**Exposures:**

Nursing homes with staff who self-confined were identified from the media and included if the confinement period of staff with residents was longer than 7 days.

**Main Outcomes and Measures:**

Mortality related to COVID-19 among residents and COVID-19 cases among residents and staff members. COVID-19 was diagnosed by primary care or hospital physicians on the basis of fever and respiratory signs (eg, cough, dyspnea) or a clinical illness compatible with COVID-19; COVID-19 diagnoses were considered confirmed if real-time reverse transcriptase–polymerase chain reaction testing for severe acute respiratory syndrome coronavirus 2 on nasopharyngeal swab was positive and considered possible if the test had not been performed or results were negative. Cases of COVID-19 were recorded by a telephone interview with the directors of nursing homes with staff who self-confined and by a nationwide declaration survey to health authorities for all facilities.

**Results:**

This study included 17 nursing homes in which 794 staff members confined themselves to the facility with their 1250 residents. The national survey included 9513 facilities with 385 290 staff members and 695 060 residents. Only 1 nursing home with staff who self-confined (5.8%) had cases of COVID-19 among residents, compared with 4599 facilities in the national survey (48.3%) (*P* < .001). Five residents (0.4%) in the nursing homes with staff who self-confined had confirmed COVID-19, compared with 30 569 residents (4.4%) with confirmed COVID-19 in the national survey (*P* < .001); no residents of facilities with self-confinement had possible COVID-19, compared with 31 799 residents (4.6%) with possible COVID-19 in the national survey (*P* < .001). Five residents (0.4%) in the nursing homes with staff who self-confined died of COVID-19, compared with 12 516 (1.8%) in the national survey (odds ratio, 0.22; 95% CI, 0.09-0.53; *P* < .001). Twelve staff members (1.6%) from the facilties with self-confinement had confirmed or possible COVID-19, compared with 29 463 staff members (7.6%) in the national survey (*P* < .001).

**Conclusions and Relevance:**

In this cohort study of French nursing homes during the COVID-19 pandemic, mortality rates related to COVID-19 were lower among nursing homes that implemented staff confinement with residents compared with those in a national survey. These findings suggest that self-confinement of staff members with residents may help protect nursing home residents from mortality related to COVID-19 and residents and staff from COVID-19 infection.

## Key Points

**Question:**

Was self-confinement of staff members with residents in French nursing homes during the coronavirus disease 2019 (COVID-19) pandemic associated with better outcomes related to COVID-19 compared with overall national outcomes?

**Findings:**

This cohort study including 17 nursing homes with staff self-confinement and 9513 nursing homes in a national survey found that nursing homes with staff self-confinement experienced lower mortality related to COVID-19 among residents and lower incidence of COVID-19 among residents and staff members than rates recorded in a national survey.

**Meaning:**

These findings suggest that self-confinement of nursing home staff members with residents may help protect residents from mortality related to COVID-19 and residents and staff from COVID-19 infection.

## Teaser

This cohort study examines coronavirus disease 2019 outcomes in nursing homes where staff self-confined with residents compared with overall national outcomes in France.

## Introduction

Coronavirus disease 2019 (COVID-19) is a devastating disease for the elderly population. The case fatality rate increases with age and is extremely high in individuals aged 80 years and older.^1,2^ Nursing home residents are a high-risk population because of their advanced age, complex medical problems, and disabilities. Nursing homes are at risk for outbreaks of respiratory infections because of the gathering of a large number of residents, health care professionals, and visitors in the facility and the high prevalence of cognitive and behavioral symptoms among residents, making it difficult to appropriately apply barriers to infection.^3^ In addition, most nursing homes are poorly prepared to implement infection control policies owing to shortage of staff and equipment and relatively low level of staff training. All these factors help to explain why COVID-19 is a major threat to nursing homes.^4^

Recently, an outbreak of COVID-19 was described in a 130-bed long-term care facility in Washington state in the US^5^ that resulted in a 78% incidence rate and a 34% case fatality rate among residents. In addition, a further 66 cases of COVID-19 in 50 staff members (incidence rate, 29%) and 16 visitors were traced to this facility.^5,6^ Health authorities^7,8^ and geriatric societies^9,10,11^ have published guidelines for long-term care facilities and nursing homes to prevent and control infection.

During the COVID-19 pandemic wave that hit France in March and April 2020, there was an initiative of some French nursing homes in which staff members decided to confine themselves to the nursing home with their residents to reduce the risk of severe acute respiratory syndrome coronavirus 2 (SARS-CoV-2), the virus responsible for COVID-19, being introduced by individuals from outside the facility.

In this cohort study, we conducted a telephonic survey to investigate the occurrence of COVID-19 diagnoses among residents and staff of these facilities and compared their data with those recorded in a national population-based survey of nursing homes conducted by French health authorities during the same period.

## Methods

The study was conducted in accordance with the principles of the Declaration of Helsinki.^12^ This study was approved by the Sorbonne University ethics committee. Owing to the retrospective design, informed consent of study participants could not be obtained and a waiver of consent was granted, per institutional policy. All the data included have been deidentified. This study is reported following Strengthening the Reporting of Observational Studies in Epidemiology (STROBE) reporting guideline.

In this retrospective cohort study, we identified French nursing homes in which some of the staff decided to confine themselves to the facility with residents on a voluntary basis for a period of 7 days or longer. We first became aware of such an initiative through newspapers and other media, and from April 20 to 24, 2020, we performed a systematic search using the Google search engine (Alphabet) to identify similar experiences. We contacted the directors of these facilities via telephone to inquire on the characteristics of the nursing home, date and duration of self-confinement of the staff, and number of cases of confirmed and possible COVID-19 that occurred from March 1 to April 28, 2020, among residents or staff, as well as the number of COVID-19–related deaths. In addition, we asked directors how staff accommodations, meals, and exceptions to confinement were managed and whether their institution had experienced any difficulties with the availability of personal protective equipment. During the telephone survey, we also identified 1 additional nursing home that had not been identified in the media as one in which staff participated in self-confinement, and we included it in the survey. We called the directors of these facilities for follow-up on June 17 and 18, 2020, to update the survey. In France, visits by relatives to residents of nursing homes were banned by the Ministry of Health from March 10 to May 11, 2020, and confinement of residents in their rooms was recommended during this period. In addition, new admissions were prohibited in the nursing homes with cases of COVID-19 among residents.

We also obtained information on the number of confirmed or possible COVID-19 cases that occurred in French facilities for elderly people using the report from Santé Publique-France, the national agency dedicated to public health, which conducts population-based national surveys on the COVID-19 pandemic in France. As a part of an investigation, directors of all French facilities for elderly people were asked to declare to Santé Publique-France the first diagnosis of confirmed or possible COVID-19 that occurred in their facility after March 1, 2020. For facilities with at least 1 COVID-19 case, directors completed a daily online questionnaire recording new cases and mortality related to COVID-19.^13,14^ Owing to the lack of information on the total numbers of facilities, residents, and staff members at the time of this national survey, we used corresponding data recorded in 2016 for French facilities for elderly people.^15,16^

The primary outcome was mortality related to confirmed or possible COVID-19 among nursing homes residents, and the secondary outcomes were cases of confirmed or possible COVID-19 among residents and staff. Possible COVID-19 was defined by the presence of fever and respiratory signs (eg, cough, dyspnea) or by a clinical disease compatible with COVID-19 according to the physician, with absence of biological confirmation of COVID-19 (no test or negative test results). Confirmed COVID-19 was defined by the presence of signs of the disease or other and a biological confirmation of COVID-19. In France, positive results on real-time reverse-transcriptase polymerase chain reaction (RT-PCR) for SARS-CoV-2 on nasopharyngeal swabs was the main test used for the biological diagnosis of COVID-19, and serological tests were not widely used until early May.

### Statistical Analysis

The number of confirmed or possible COVID-19 cases in nursing homes with staff who self-confined was compared with that obtained from the national survey using odds ratios (ORs) and their 95% CIs, χ^2^ test, and Fisher exact test. *P* values were 2-sided, and *P* < .05 was considered statistically significant. Stata statistical software version 13 (StataCorp) was used for all statistical calculations.

## Results

### Nursing Homes With Staff Confinement and Their Characteristics

We identified 17 nursing homes that implemented voluntary staff confinement with residents, with 794 staff members and 1250 residents. Their characteristics are shown in Table 1. These facilities had diverse legal statuses and were located in a variety of geographic areas, including areas where there was a high overall mortality rate from COVID-19 (Table 1; eFigure in the Supplement). Thirteen facilities (76.5%) experienced shortages of personal protective equipment (eTable in the Supplement).

**Table 1.** Characteristics of Nursing Homes With Staff Self-confinement Periods of Staff Members With Residents

Characteristic	Nursing homes, No. (%) (N = 17)
Type	
Public	6 (35.3)
Private	4 (23.5)
Nonprofit	7 (41.2)
Size, residents, No.	
≤60	6 (35.3)
61-100	7 (41.2)
>100	4 (23,5)
Geographic area	
By overall mortality, deaths per 100 000 population	
≤9	6 (35.3)
10-24	8 (47.1)
≥25	3 (17.6)
By incidence of COVID-19 in nursing homes, % of residents^a^	
<3	8 (47.1)
3-5.9	1 (5.9)
6-10	5 (29.4)
>10	3 (17.6)
Residents, mean (SD), No.	73.5 (27.6)
Staff members, mean (SD), No.	46.7 (22.7)
Staff who participated in confinement, mean (SD), No.	32.7 (7.3)
Duration of first confinement period, d	
11	1 (5.9)
14-15	11 (64.7)
21	4 (23.5)
28	1 (5.9)
Participated in a second confinement period	5 (29.4)
Duration of second confinement period, d	
14-15	4 (23.5)
21	1 (5.9)
Precautions for staff prior confinement	
Monitor temperature 2 times/d and inquire on symptoms	17 (100)
Swab SARS-CoV-2 RT-PCR testing before first period	3 (17.6)
Swab SARS-CoV-2 RT-PCR testing before second period	4 (23.5)
Main reason for not renewing confinement	
Insufficient volunteers	6 (35.3)
Implementation of testing of residents and staff	3 (17.6)
End of predefined duration project	8 (47.1)
Administrative injunction	2 (11.8)
Early termination owing to administrative injunction	1 (5.9)

Abbreviations: COVID-19, coronavirus disease 2019; RT-PCR, reverse transcriptase–polymerase chain reaction; SARS-CoV-2, severe acute respiratory syndrome coronavirus 2.

^a^ Regions with high COVID-19 incidence in nursing homes: Grand-Est, Île-de-France; intermediate COVID-19 incidence: Auvergne-Rhône-Alpes, Bourgogne-Franche-Comté, Hauts-de-France; low COVID-19 incidence: Centre-Val de Loire; Provence-Alpes-Côte d’Azur; very low COVID-19 incidence: Bretagne, Normandie, Nouvelle Aquitaine, Pays de la Loire, Occitanie.

### Features of Staff Confinement With Residents

During self-confinement periods, all participating staff members remained in the nursing home 7 days a week and 24 hours a day. Staff members who self-confined included nurses, nurses aids, directors and their assistants, housekeeping staff, kitchen staff, and activities workers, as well as a psychologist, a physician, and an occupational therapist. During self-confinement periods, staff members slept in various unused areas of the nursing home (eg, offices, rehabilitation rooms, dining rooms) using extra beds or camping beds. In 3 nursing homes, there were also mobile homes available in the parking lot of the facility for daytime rest for staff working at night. The time courses of the self-confinement periods are shown in the Figure. Twelve nursing homes had a single course of self-confinement, and 5 nursing homes started a second course of self-confinement immediately after the first with renewed staff. At the time of the survey, 4 nursing homes were still in their second self-confinement period; the directors of nursing homes with self-confinement were contacted again on June 17 to 18, 2020, to check if new cases of COVID-19 were recorded in their facilities. In all 17 nursing homes, the temperature of all the staff members was measured 2 or 3 times a day, and symptoms of COVID-19 were queried daily; all staff members who participated in the self-confinement initiative did not have fever or respiratory symptoms. In 3 nursing homes, screening for COVID-19 via nasal swab RT-PCR testing was performed among all the staff members who volunteered to participate before beginning the self-confinement periods; in 2 other nursing homes, the screening by RT-PCR testing was conducted among staff members only for the second period of self-confinement (Figure). There were few exceptions to the interdiction against entering and leaving the institution during self-confinement periods. Of course, physicians and ambulance attendants were permitted to enter and leave the facility for medical consultations or to drive a patient to and from the hospital. A few family visits were allowed in end-of-life situations as well as the arrival of funeral staff for body lifts. For 5 facilities with meal deliveries, the delivery personnel did not enter the premises. No nursing home with staff who practiced self-confinement had new admissions of residents during the self-confinement periods.

**Figure.**
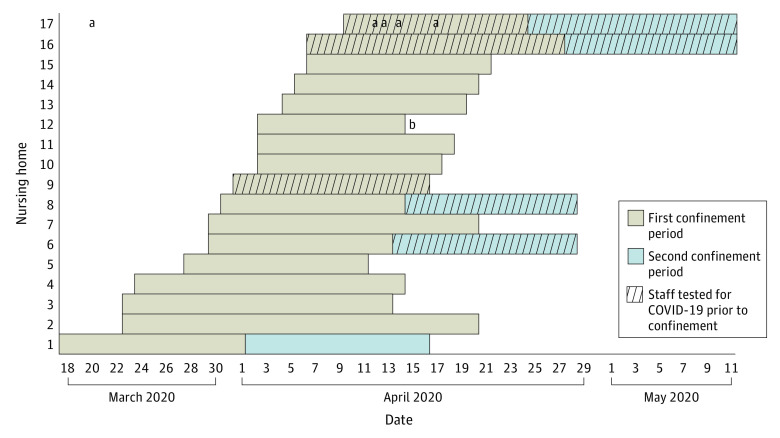
Staff Confinement Periods With Residents in Each of the 17 Nursing Homes Coronavirus disease 2019 (COVID-19) testing was performed via reverse transcriptase–polymerase chain reaction tests for severe acute respiratory syndrome coronavirus 2 on nasal swabs. ^a^Represents the onset of a coronavirus disease 2019 case in the nursing home. ^b^This facility terminated the self-confinement period early owing to administrative injunction.

### Mortality Related to COVID-19

The national survey included a total of 9513 facilities, 695 060 residents, and 385 290 staff members (Table 2). The May 7, 2020, report of the national survey recorded 4599 facilities with at least 1 case of COVID-19 and 12 521 deaths related to COVID-19.

**Table 2.** Outcomes in Facilities With Staff Confinement With Residents and in the Population-Based Facilities Surveyed by Health Authorities

Outcome	No. (%)		Odds ratio (95%CI)	*P* value
	With self-confinement	National survey		
Facilities				
Total, No.	17	9513	NA	NA
With ≥1 COVID-19 case among residents	1 (5.8)	4599 (48.3)	0.07 (0.01-0.50)	<.001
Residents				
Total, No.	1250	695 060	NA	NA
Cases of confirmed COVID-19^a^	5 (0.4)	30 569 (4.4)	0.09 (0.04-0.21)	<.001
Cases of possible COVID-19	0	31 779 (4.6)	NA	<.001
Total cases of COVID-19	5 (0.4)	62 368 (9.0)	0.03 (0.02-0.10)	<.001
Deaths related to COVID-19	5 (0.4)	12 516 (1.8)	0.22 (0.09-0.53)	<.001
Staff members				
Total, No.	794	385 290	NA	NA
Cases of confirmed COVID-19^a^	6 (0.8)	14 645 (3.8)	0.19 (0.09-0.43)	<.001
Cases of possible COVID-19	6 (0.8)	14 806 (3.8)	0.19 (0.09-0.43)	<.001
Total cases of COVID-19	12 (1.6)	29 451 (7.6)	0.19 (0.10-0.33)	<.001

Abbreviations: COVID-19, coronavirus disease 2019; NA, not applicable.

^a^ Confirmed COVID-19 was defined as a positive result for severe acute respiratory syndrome coronavirus 2 with reverse transcription–polymerase chain reaction testing.

At the time of investigation, only 1 facility with self-confinement (5.8%) had cases of COVID-19 among the residents, compared with 4599 facilities (48.3%) in the national survey (*P* < .001) (Table 2). Among facilities with self-confinement, 5 residents (0.4%; all in the same facility) had confirmed COVID-19, compared with 30 569 residents (4.4%) with confirmed COVID-19 in the national survey (*P* < .001); no residents of facilities with self-confinement had possible COVID-19, compared with 31 799 residents (4.6%) with possible COVID-19 in the national survey (*P* < .001). A total of 5 residents (0.4%) in facilities with self-confinement died of COVID-19, compared with 12 516 residents (1.8%) in the national survey who died of COVID-19 (OR, 0.22; 95% CI, 0.09-0.53; *P* < .001). Among nursing homes with self-confinement, 6 staff members (0.8%) had confirmed COVID-19 (none of these staff members participated in self confinement), compared with 14 645 staff members (3.8%) in the national survey (*P* < .001); similarly, 6 staff members (0.8%) in facilities with self confinement had possible COVID-19 (only 1 of these staff members participated in self-confinement), compared with 14 806 staff members (3.8%) in the national survey (*P* < .001) (Table 2).

### Case Report

Nursing home No. 17, in which cases of COVID-19 occurred among residents, is located in a French region that was heavily affected by the pandemic. One resident developed COVID-19 symptoms on March 20, before the facility decided to organize a period of self-confinement. The patient was referred to a hospital, and the diagnosis of COVID-19 was confirmed by a positive RT-PCR test for SARS-CoV-2; the resident came back to the nursing home after 2 days. This event and the regional context led the staff members to propose self-confinement to minimize to virus circulation associated with staff displacements. All staff who volunteered to participate in the first self-confinement period were screened for SARS-CoV-2 via nasal swab testing and RT-PCR. Three staff members were found to be asymptomatic carriers and were excluded from the self-confinement initiative. The same procedure was performed before the second self-confinement period, and all tests had negative results. During the first self-confinement period, 4 additional residents developed COVID-19 confirmed by positive RT-PCR testing, all of whom were residing in the same unit as the first resident diagnosed with COVID-19. The onset date of the cases is shown in Figure. All 5 residents died.

## Discussion

This cohort study found that nursing homes in which staff decided to remain confined to the facility with the residents experienced a significantly lower incidence of COVID-19 among the residents and lower mortality than did those included in the national survey conducted during the same period. The number of cases of COVID-19 among staff members of facilities with self-confinement was also significantly lower than that reported in the national survey.

All but 1 of the nursing homes remained free of cases of COVID-19 among residents, indicating that in these facilities, staff members fulfilled their common aim to block entry of SARS-CoV-2 into the facility and prevent COVID-19–related mortality. In these facilities, the directors ensured that no staff members had symptomatic COVID-19 before initiating self-confinement; however, screening for asymptomatic SARS-CoV-2 carriers was performed in only a small number of facilities. The presence of an asymptomatic carrier among the staff confined with the residents could have been a source of an outbreak in the facility despite their efforts.

However, nursing home No. 17 experienced a very different situation, as a confirmed case of COVID-19 was diagnosed before the staff confinement period. The purpose of the self-confinement initiative was less clear in this facility, as SARS-CoV-2 was already circulating there, accounting for 4 additional cases of COVID-19 among residents.

### Limitations

This study has several limitations. It is impossible to assert a causal link between the self-confinement initiative and the low incidence of COVID-19. The identification of these initiatives from newspapers and other media is probably not a strong bias, as all were reported by journalists shortly after the start of the confinement of the staff and not on the basis of the outcomes of the residents. The type and size of the nursing homes varied, and most were located in regions of France affected by the pandemic.^13,14^ To the extent that the containment experience of staff reflects exceptional dedication to their residents, it may be associated with improved hygiene practices. Another limitation is related to the recording of COVID-19 cases in the study. The definition of COVID-19 cases was similar in our telephone survey and in the national survey,^13^ but the latter may have missed some cases of COVID-19 owing to underreporting by some nursing homes directors. In addition, some cases of COVID-19 may have been missed in both surveys owing to atypical clinical presentations or negative RT-PCR test results, but this was probably not common in the nursing homes with self-confinement owing to the very high level of awareness about COVID-19 among staff members.

## Conclusions

The findings of this cohort study suggest that the initiative of staff members to confine themselves to nursing homes with residents was successful in protecting their facilities from an outbreak of COVID-19. These experiences, which are based on a strong voluntary investment of staff, including managers, cannot be presented as a generalizable model because of several barriers, such as low sustainability, labor law regulations, and consequences on staff family life. However, in the context of a pandemic threat, staff members in a nursing home free from COVID-19 cases who have decided to confine themselves to the nursing home should not be discouraged; furthermore, screening for asymptomatic SARS-CoV-2 infections should be offered to staff members before they begin the confinement period with residents to promote better outcomes.

## Notes

Supplement. **eTable.** Occurrence and Duration of Shortages of Personal Protection Equipment From March 1 to May 11, 2020, in Nursing Homes Where Staff Members Self-Confined With Residents **eFigure.** Locations of Nursing Homes With Staff Who Self-Confined With Residents Stratified by Regional Incidence of COVID-19 Among Nursing Home Residents
