# Supplementary material for: Coronavirus Disease 2019 Outcomes in French Nursing Homes That Implemented Staff Confinement With Residents
Source: JAMA Netw Open. 2020 Aug 13;3(8):e2017533. doi: 10.1001/jamanetworkopen.2020.17533 (PMC7426749; doi:10.1001/jamanetworkopen.2020.17533)
Supplement: Supplement. — eTable. Occurrence and Duration of Shortages of Personal Protection Equipment From March 1 to May 11, 2020, in Nursing Homes Where Staff Members Self-Confined With Residents eFigure. Locations of Nursing Homes With Staff Who Self-Confined With Residents Stratified by Regional Incidence of COVID-19 Among Nursing Home Residents [file jamanetwopen-3-e2017533-s001.pdf]

Supplementary Online Content

Belmin J, Um-Din N, Donadio C, et al. Coronavirus disease 2019 outcomes in French nursing homes that implemented staff confinement with residents. *JAMA Netw Open.* 2020;3(8):2017533. doi:10.1001/jamanetworkopen.2020.17533

eTable. Occurrence and Duration of Shortages of Personal Protection Equipment From March 1 to May 11, 2020, in Nursing Homes Where Staff Members Self-Confined With Residents
eFigure. Locations of Nursing Homes With Staff Who Self-Confined With Residents Stratified by Regional Incidence of COVID-19 Among Nursing Home Residents

This supplementary material has been provided by the authors to give readers additional information about their work.

© 2020 Belmin J et al. *JAMA Network Open.*

eTable. Occurrence and Duration of Shortages of Personal Protection Equipment From March 1 to May 11, 2020, in Nursing Homes Where Staff Members Self-Confined With Residents

	Shortage of		
	medical mask	alcohol based rub	disposable clothing
None	4 (24%)	4 (24%)	4 (24%)
During 1-2 weeks	4 (24%)	4 (24%)	0
During 3-4 weeks	2 (12%)	0	0
During 5-6 weeks	5 (29%)	6 (35%)	0
During 7-8 weeks	1 (6%)	0	6 (35%)
During 9-10 weeks	1 (6%)	3 (18%)	7 (41%)

© 2020 Belmin J et al. *JAMA Network Open.*

eFigure. Locations of Nursing Homes With Staff Who Self-Confined With Residents Stratified by Regional Incidence of COVID-19 Among Nursing Home Residents

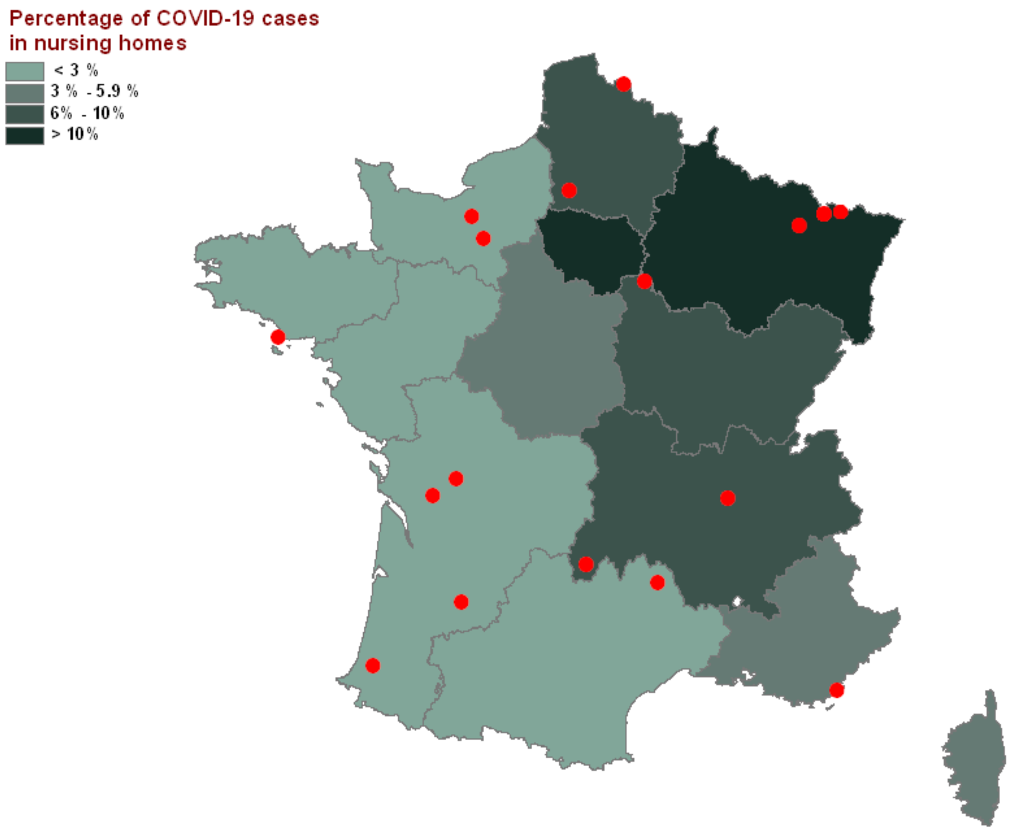

© 2020 Belmin J et al. *JAMA Network Open.*
